# Preventing Depression in Adolescents With Depressed Parents: Insights From Randomized Controlled Trials and Parenting Interventions

**DOI:** 10.7759/cureus.87314

**Published:** 2025-07-05

**Authors:** Dhrubajyoti Bhuyan, Ishmita Paul, Nikhita Das, Monalisa Boro, Vivek Saharan, Lakshi Raina

**Affiliations:** 1 Psychiatry, Assam Medical College & Hospital, Dibrugarh, IND; 2 Trauma and Orthopaedics, The Hillingdon Hospitals, London, GBR; 3 Psychiatry, All India Institute of Medical Sciences, Guwahati, Guwahati, IND

**Keywords:** children, emotional and behavioral disorders, parenting, parent management training (pmt), prevention of depression

## Abstract

Childhood abuse in adolescents has long been linked to an elevated likelihood of harmful consequences in both adolescence and adulthood. Externalizing behavior disorders, such as attention deficit hyperactivity disorder (ADHD), are among the most common reasons children and adolescents are referred for mental health care. Another common illness among children and teenagers is depression. Among the principal risk factors for young people acquiring depression is parental depression. Besides genetic heredity, a variety of changeable psychological and social variables, such as a negative thinking style, paternal emotions, and uneven parenting behaviors, might increase the likelihood of depression in this population. The development of preventative therapies aimed at addressing these health risks has generally focused on psycho-educational components, teaching kids regarding parental depression and/or cognitive-behavioral components meant to boost their psychological fortitude. The initial effects of preventive therapies for adolescents with depressed parents tend to be strongest immediately after intervention but may diminish over time. Treatments created to break this loop offer help and knowledge to expectant mothers who are more likely to face challenges with parenting.

## Introduction and background

To assess the effectiveness of an intervention and determine whether an ecological factor significantly impacts childhood health, a randomized controlled trial (RCT) is recommended. The intervention should aim to: improve access to perinatal and pediatric care, enhance knowledge of newborn development, strengthen parent-infant bonding, expand the availability of psychological services, prevent repeat pregnancies, and support mothers in improving their overall life trajectories. Many empirical studies back different Cognitive Behavioral Therapy (CBT) regimens for externalizing issues in children and teenagers. Results from multiple RCTs have been used to support various treatment strategies for externalizing disorders. Due to the high prevalence and poor prognosis of disruptive conduct in preschool-aged children, this issue becomes a matter of great concern. According to potential mediating studies, variations in mother-child relations, and good and unfavorable parenting behaviors could be used to explain changes in child behavior [1]. Our culture is increasingly concerned about behavioral issues in children, which are linked to a variety of negative life events [2]. While multiple RCTs support CBT for externalizing behaviors in children, limited research exists on how ecological interventions, such as maternal life trajectory and parent-infant bonding, interact with these treatments. Future studies should integrate environmental and psychological variables in trial designs to evaluate their combined effect on child behavior outcomes and identify key mediating parenting behaviors.

## Review

Child abuse is a widespread issue, and parents who experienced it themselves as children may mistreat their children when they become parents [3]. This article aims to provide a concise review of the RCTs evaluating the effectiveness of interventions to prevent child abuse.

The link between parental depression and a heightened risk of depression in adolescents is widely recognized in psychological literature. Research consistently indicates that teens with parents who suffer from depression are more susceptible to developing depressive symptoms. For example, a study featured in the Asian Journal of Social Health and Behavior reported a significant correlation between parental and adolescent depression in an Indonesian population [4]. Anxiety and depression in children raise the likelihood of developing severe depression and suicidal behavior as adults, in addition to being linked to brief psychological, academic, and societal factors. A subgroup analysis explored whether certain preventive measures are more effective than others, despite most strategies being evaluated in only a single study. This meta-analysis showed that preventive therapies are effective for children whose parents are depressed [5].

The initial preferred treatment for adolescent depressive disorders is CBT; however, there is room for advancement given the therapy's low overall success. Building a robust evidence base for these key variables can help tailor CBT to individual children and adolescents, potentially reducing the risk of using ineffective or harmful therapeutic components. Although 65% of other aspects of CBT were not deemed to be beneficial by children and adolescents, behavioral activation and challenging thoughts were seen as a feasible and useful technique in lowering depressive symptoms [6].

Adolescents with externalizing disorders perform less well academically and socially than their peers and are more likely to experience adverse adult outcomes, such as criminal activity, unemployment, financial troubles, and in some cases, higher risks of adverse outcomes in their offspring, including neonatal mortality. Additionally, they are more likely to experience emotional issues due to the high rates of comorbidity between externalizing behavior problems and both depression and anxiety in youngsters. This appears to cause greater disability than when toddlers are identified with these disorders separately. According to research, cognitive and psychological issues can be detected as early as the age of two years, and up to one in five young kids struggle with these issues. Children living in poverty are more likely to encounter these issues than other children [7,8].

In order to lessen conduct issues, researchers have also employed a variety of parenting techniques that emphasize developing strong parent-child bonds as well as child behavior management techniques. Aggressive parenting was assessed using the Responsible Adult Dispute Strategies Index. One of the first studies to assess the early findings of a parenting program in decreasing the risk of childhood neglect in families with kids aged three to eight used an RCT assessment. The intervention was successful in enhancing positive parenting, but it may have had an unintended adverse effect on the conduct of the children [9].

The significant cost of children's mental health issues to society and those impacted makes them a priority for treatment. Difficulties in emotional control are a common underlying factor that contribute to the development and continuation of many internalizing and externalizing disorders. Adopted children and adolescents are more likely than their non-adopted peers to experience emotional, behavioral, cognitive, and relationship difficulties. They are also more frequently referred to mental health services due to these challenges [1].

Luby et al. (2018) demonstrated that anxiety and depression can be reliably identified in children as young as three years old, with prevalence rates comparable to those observed in school-aged children. This RCT of parent-child psychoanalysis for early childhood depression highlights a promising new direction for treatment: early recognition and intervention for this persistent and recurrent condition. Since school-age depression has proven difficult to treat effectively through regulation-based approaches alone, there is a clear need to develop and test early interventions. To accurately evaluate the treatment's effectiveness, children currently on antidepressants or undergoing ongoing psychotherapy were excluded from the study [6].

Methodology

A structured literature review was carried out using electronic databases such as PubMed, PsycINFO, and Google Scholar, targeting peer-reviewed publications from 2015 to 2023. Studies were included based on the following criteria: (1) participants aged 0 to 20 years; (2) focus on internalizing or externalizing psychological disorders; (3) assessment of interventions including CBT, Parent Management Training (PMT), Emotion-Focused Therapy, or programs enhancing parental psychological flexibility; and (4) the use of RCTs or other rigorous empirical designs.

Each selected study was reviewed for participant demographics, age groups targeted, clinical concerns addressed, type of therapeutic intervention used, and outcome measures. Particular attention was given to how parental involvement, psychological adaptability, and developmental stage influenced treatment outcomes. Findings were synthesized in a comparative table to highlight patterns across different therapeutic models and age brackets. Rather than assigning numeric quality scores, the methodological strength and potential limitations of each study were assessed descriptively. Table 1 summarizes the findings.

**Table 1:** Age-related emotional and behavioral disorders in children and adolescents PMT: Parent Management Training; CBT: Cognitive Behavioral Therapy; RCT: Randomized Controlled Trials; DEF: Dvelopmental education for families

Author and year of publication	Focus of the study	Sample characteristics (age)	Symptoms addressed	Intervention/Treatment	Key findings
Sukhodolsky et al. (2016) [10]	Behavioral treatments for children and adolescents with angry, anxiety, and aggressive behavior	1-4 years old and adults over the age of 18	Violent outbreaks and aggressive behavior in children are one of the most common causes of recommendations for inpatient mental health care. The treatment goals of behavioral therapies for irritability, anger, and aggression	PMT to improve negative family interactional patterns that lead to disruptive conduct in kids. CBT to target aggressive behavior by addressing deficits in problem-solving, social skills, and emotional regulation.	PMT and CBT have been thoroughly researched via RCTs in adolescents with disrupted behavior problems, whereas investigations of pathophysiologic CBT for anger and aggressiveness are currently ongoing.
Levey et al. (2017) [11]	Child abuse and neglect	Above six months to 15 years	Abused adolescents are more likely to develop medical and mental health issues as adults, especially post-traumatic depressive illness, inflammatory process disease, asthmatic issues, drug and alcohol addiction, depression, and suicidal tendencies.	Maternity home visitation intervention; there were eight distinct RCTs aimed at reducing child abuse.	All RCTs of interventions aimed at preventing maltreatment amongst high-risk mothers were included in this analysis. These studies collectively showed a significant reduction in reported cases of child maltreatment and improved maternal sensitivity.
Loechnera et al. (2018) [12]	Being a depressive parent is a significant risk factor for developing depression	18 years or younger	Both the frequency and intensity of adolescent depression served as both major and supplementary outcome indicators.	DEF was used as a randomized control experiment	Minor but substantial post-intervention preventative benefits on reducing depressive/internalizing signs as well as modest to medium protective impact on the occurrence of depression were found.
Ouda et al. (2019) [13]	Depression as another common disease in youngsters and adolescents.	Children and adolescents between the ages of 15 and 20 years	The heightened chance of suicide, the emergence of behavioral issues, inappropriate medication use, subpar academic achievement, and poor social interaction are all associated with depression.	CBT	Although CBT is the first-line treatment for symptoms of teenage depression and manifestations, there is still room for improvement. This research suggests that when behavioral activation and challenging ideas are included in CBT, together with the involvement of the caregiver(s), the outcomes may be improved.
Flujas-Contreras et al. (2020) [14]	The efficacy of a content treatment for improving parental emotional control and psychological adaptability.	Children and adolescents between the ages 10 and 18 years	Anti-democratic parental involvement can result in problems with internalization and externalization as well as less pragmatic behavior in children.	An informed contextual therapy parenting program called "Parenting Forest" aims to help parents regulate their emotions and increase their psychological flexibility.	The parents' emotional control and psychological adaptability were positively impacted, according to the findings.
Riise et al. (2021) [15]	Diseases that externalize in children and adolescents	Children and adolescents between 15 and 20 years	Children and adolescents with externalizing behavior problems, such as Attention Deficit Hyperactivity Disorder (ADHD), Conduct Disorder (CD), and Oppositional Defiant Disorder (ODD), are frequently referred to mental health treatment.	A large body of empirical research supports a variety of CBT treatments for externalizing issues in both children and adolescents.	The results demonstrate that CBT for externalizing disorders can be transferred from academic settings to ordinary clinical care.
Ansar et al. (2022) [8]	Emotion-focused parenting programs to help parents understand and manage their child's symptoms and perceived stress during childhood.	Children and adolescents between 6 and 13 years of age	The significant cost of children's mental health issues to society and those impacted makes them a recognized major priority treatment focus.	A 12-week parenting program called Emotion-Focused Skills Training (EFST), which is based on emotion-focused therapy, was created to help children and adolescents with their mental health issues.	For children and adolescents with internalizing and externalizing symptoms, EFST showed effectiveness in reducing pain.

CBT for anger, irritability, and aggression

Anger is a negative state of feeling that can result in greater bodily stimulation, emotions of guilt, and a developmental outcome for aggressive behavior. Disappointment or psychological retaliation are frequent causes of anger. It can last for minutes or seconds, and its strength can be anything from mild irritation to hatred and violence. Investigations using confirmatory factor analysis differentiate between internal feelings of anger (i.e., angry perception) and external displays of anger (i.e., an individual's propensity to show outrage in these ways, repress it, or start managing it). Child-directed CBT techniques that teach techniques for coping with anger and frustration as a part of a larger repertoire of emotion regulation strategies place a major emphasis on enhancing the ability to control anger [10].

From a constructivist perspective, different facets of the feeling and representation of rage appear at various ages and proceed along various developmental paths. Children aged one to four years old frequently have childish tantrums that also include yelling, screaming, dragging, punching, and striking. These episodes are approximately five to ten minutes in length and occur five to nine times per week. Even though growing adolescents usually continue to openly express their anger and irritation, which parents frequently mistake for tantrums, the severity and frequency of tantrums tends to decrease with age. The definition of aggression is an overt action that may cause harm to oneself or others [14].

Sleep problems

Adolescents often struggle with sleep disturbances, which can have a significant impact on their behavior and emotional well-being. These sleep problems are commonly associated with behavioral and emotional challenges in children. Moreover, CBT aims to help parents understand the influence of their behaviors on their child’s sleep patterns and guide them on how to modify these behaviors effectively [16].

Parent management training (PMT) concepts and effectiveness

The physiological, ecological, and behavioral health issues that combine to cause adolescent frustration and violent behavior that result in tantrums, aggression, and disobedience are addressed to improve family patterns of communication. The essential idea behind operant conditioning, which forms the basis of PMT, is that the probability of a behavior repeating depends on the events that occur after it. It has also been demonstrated that forceful and uneven supervision, such as excessive reprimanding and physical punishment, increases aggressive tendencies. By enhancing parenting competency in handling these disruptive behaviors, PMT aims to minimize the child's hostility and disobedience. Parents undergoing PMT are taught to recognize the purpose of disruptive conduct, compliment good behavior, effectively convey instructions, ignore disruptive attention-seeking behavior, and employ consistent punishments for it. While some approaches involve children while practicing new parenting techniques, PMT is conducted exclusively with the parents [10].

Strategies for child-directed CBT

CBT is designed to reduce aggressive behavior by improving deficits in emotional regulation and social problem-solving skills. The term 'cognitive behavioral intervention' is used to describe treatments carried out with children and emphasizes the application of structured procedures and learning principles to bring about changes in thinking, feeling, and behavior. Common strategies to address aggressive behavior include identifying its causes and effects, learning to recognize and manage anger, and using interpretation, empathy, and cognitive restructuring techniques. Additionally, individuals are taught to model and practice socially acceptable behaviors as alternatives to angry or aggressive responses. Even though CBT is carried out with youngsters, parents play a variety of roles in the process, including transporting the children to therapy, informing other family members about the child's behavioral issues, and setting up a place for the child to practice CBT skills between appointments [13].

Modulation of the child's hypothalamic-pituitary-adrenal (HPA) axis and parental involvement

While unfavorable childhood experiences may lead to aberrant nocturnal cortisone release, fast and flexible reactions from parents in response to their child's cues may encourage the development of appropriate regulating mechanisms in children. Adolescents who are adopted abroad frequently go through institutional care and other types of adversity that might change how their HPA axis functions. Compared to children nurtured by their birth parents, adolescents who have been adopted internationally typically have less pronounced diurnal reductions. Infants and young children have a vital necessity to establish intimate connections with a select group of adult custodians who look after them consistently [5].

Parenting classes

There is an urgent demand for parenting programs that can be widely distributed in environments with limited resources. The prevention of violence against children has been linked to such programs. The primary outcomes were parental and child behaviors, assessed from multiple outcomes. Many scientifically validated programs, particularly parenting interventions, have been found to be effective in low- and middle-income country settings. These programs are recognized as an efficient method for decreasing violence towards children and the aggression occurring later in children [17]. The foundation of children and adolescent mental health prevention and therapy is parental intervention. Parenting strategies that are emotionally centered work to improve parent-child relationships and promote positive emotional interaction to nurture children's full emotional potential. Emotion-focused therapy helps parents focus on emotions rather than behavior to strengthen their relationship and improve communication [18].

Developmental education for families (DEF)

A scientifically proven, home-visiting program created to enhance children's mental and linguistic development serves as the model for the DEF program. With the aid of both physical and occupational specialists, the DEF program was altered to accommodate the unique needs of globally adopted children. Parent educators concentrated on strategies to support children in achieving developmental stages using play activities and by giving them a chance to practice. Depending on the child's developmental phase, specific activities were selected. The parents' abilities were reviewed using video feedback as well. To distinguish it from Attachment and Biobehavioral Catch-up (ABC), parental sensitivity-related elements were left out [19]. 

Yoga decreased depressive and anxiety symptoms

Another review sought to assess yoga's application and efficiency in easing anxiety and depressive symptoms in young people. The goal of yoga, a multidimensional ancient practice, is to improve quality of living through the fusion of the physique and mind with a focus on self-awareness. It has been suggested that yoga, as a comprehensive tradition rooted in the practice of physical postures, has both physical and mental advantages. The use of yoga to treat adolescent mental health disorders has led to the publication of numerous research studies on the topic [20].

## Conclusions

Compared to adolescents who engaged in an active or non-active controlled group, adolescents of depressed parents who engaged in a selective preventative intervention exhibited fewer depressive symptoms and were less likely to receive a diagnosis of depression in the future. Home visiting, which was initially created to enhance the health of preterm births, has since been applied to treat postnatal depression, enhance parent-infant bonding, reduce child maltreatment, and enhance child development.

Future research should assess the needs of women who have experienced childhood abuse and identify effective interventions to support their well-being. As a result, the present article's use of an RCT methodology and the absence of base inequalities between adolescents placed in the different interactive conditions strengthen the certainty that interventions such as CBT, PMT, and other behavioral therapies are effective. By using a preventive and comprehensive strategy, early diagnosis may enhance children's emotional and behavioral health.
